# Distolingual root prevalence in mandibular first molar and complex root canal morphology in incisors: a CBCT analysis in Indian population

**DOI:** 10.1038/s41598-024-51198-1

**Published:** 2024-01-03

**Authors:** Komal Sheth, K. S. Banga, Ajinkya Pawar, Dian Agustin Wahjuningrum, Mohmed Isaqali Karobari

**Affiliations:** 1 Department of Conservative Dentistry and Endodontics, Nair Hospital Dental College, Mumbai Central, Mumbai, 400 008 India; 2 Department of Conservative Dentistry, Faculty of Dental Medicine, Universitas Airlangga, Surabaya, East Java Indonesia; 3 Department of Restorative Dentistry and Endodontics, Faculty of Dentistry, University of Puthisastra, Phnom Penh, 12211 Cambodia; 4 Dental Research Unit, Center for Global Health Research, Saveetha Medical College and Hospital, Saveetha Institute of Medical and Technical Sciences, Saveetha University, Chennai, Tamil Nadu 600077 India

**Keywords:** Dentistry, Dental pulp

## Abstract

Cone-beam computed tomography was used to understand the possible correlation between the prevalence of distolingual root (DLR) in permanent mandibular first molars (MFMs) and the associated complicated mandibular incisor’s root canal morphology (MIs) in an Indian population. A total of 400 scans were evaluated for MFMs and MIs. The prevalence of DLRs and root canal anatomy of MIs were assessed based on Vertucci’s classification, and then the sample were grouped according to age, sex and side. Statistical analysis was used to evaluate the possible correlation between the presence of DLRs in the first molar and root canal morphology of incisors. Chi square test was used to evaluate the correlation between the root canal configurations of MIs with the existence of DLRs in MFMs. There was no statistically significant difference between sexes or ages for the prevalence of DLRs in the first molars (*p* > 0.05), which was 6.62%, with the right side having a greater frequency of DLRs (7.8%) than the left (5.5%). Vertucci Type I canal configuration was most common for the mandibular central (66.75%) and lateral incisors (58.62%). Vertucci Type III was the most common complicated canal morphology, followed by Types V, II, and IV for MIs, with no statistically significant difference in the studied sample's age and sex. (*p* < 0.05). No association was observed between the presence of DLRs in first molars and complicated root canal configurations in MIs. Taken together, the possibility of complicated root canal configuration in MIs was lesser in the presence of DLRs in MFMs among the Indian population.

## Introduction

Knowledge of tooth and root canal anatomy is crucial for dental practise and for recognising anthropologically significant traits^1^. Variations in root number and canal morphology are common in permanent mandibular first molars (MFMs), despite the fact that they typically have two roots positioned mesially and distally having three root canals. Many anatomical variations in the roots and root canals of MFMs have been suggested^2,3^. A major anatomical variation in two-rooted MFMs is the occurrence of an additional root distolingually or mesiobuccally, known as radix entomolaris (RE) or radix paramolaris, respectively, as first reported by Carabelli^4^.

Speculation surrounds the reasons for of RE formation. Curzon suggested that the "three-rooted molar" characteristic has a high level of genetic penetrance; this dominance was demonstrated by the occurrence of the trait in both pure Eskimo and Eskimo/Caucasian mixes^5^. According to anatomical studies, some ethnic groups—including populations with Mongoloid features like Chinese, Korean, American Indians, Taiwanese and Eskimo, Korean —have distinct DLRs in their first mandibular molars^5–12^.

**Figure 1:**
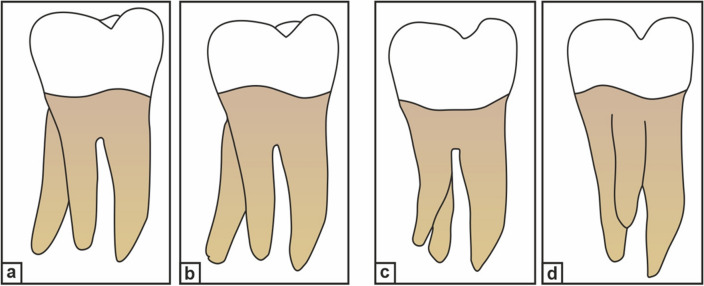
Carlsen and Alexandersen’s classification of radix entomolaris (RE) based on its location of cervical part. (**a**) Type A: the RE is located lingually to the distal root complex which has two cone-shaped macrostructures. (**b**) Type B: the RE is located lingually to the distal root complex which has one cone-shaped macrostructures. (**c**) Type C: the RE is located lingually to the mesial root complex. (**d**) Type AC: the RE is located lingually between the mesial and distal root complexes.

**Figure 2:**
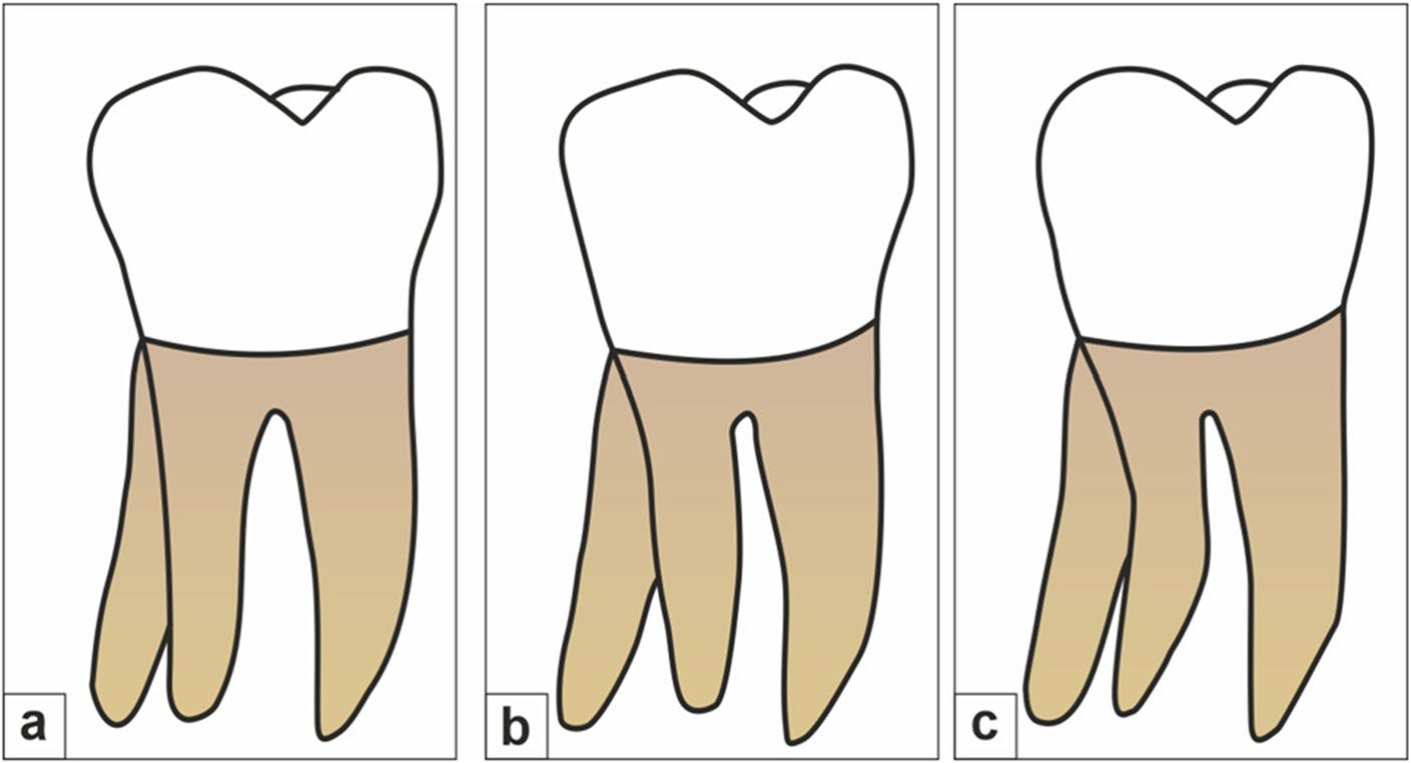
Ribeiro and Consolaro’s classification of Radix entomolaris based on its bucco-lingual orientation. (**a**) Type 1: a straight root or root canal. (**b**) Type 2: a curved coronal third which becomes straighter in the middle and apical third. (**c**) Type 3: an initial curve in the coronal third with a second buccally oriented curve which begins in the middle or apical third.

Carlsen and Alexandersen^4^ (Fig. 1), Ribeiro and Consolaro^13^ (Fig. 2), and Wang et al.^14^ have classified RE based on its morphology, buccolingual orientation, and radiographic appearance, respectively. The complexity of the DLR, such as the presence of narrow canals, buccolingual curvature, and short root length, makes biomechanical preparation and obturation of the canal difficult. Moreover, position and superimposition of the DLR over the main distal root may lead to failure in identifying and preparing it, resulting in the failure of endodontic treatment.

**Figure 3:**
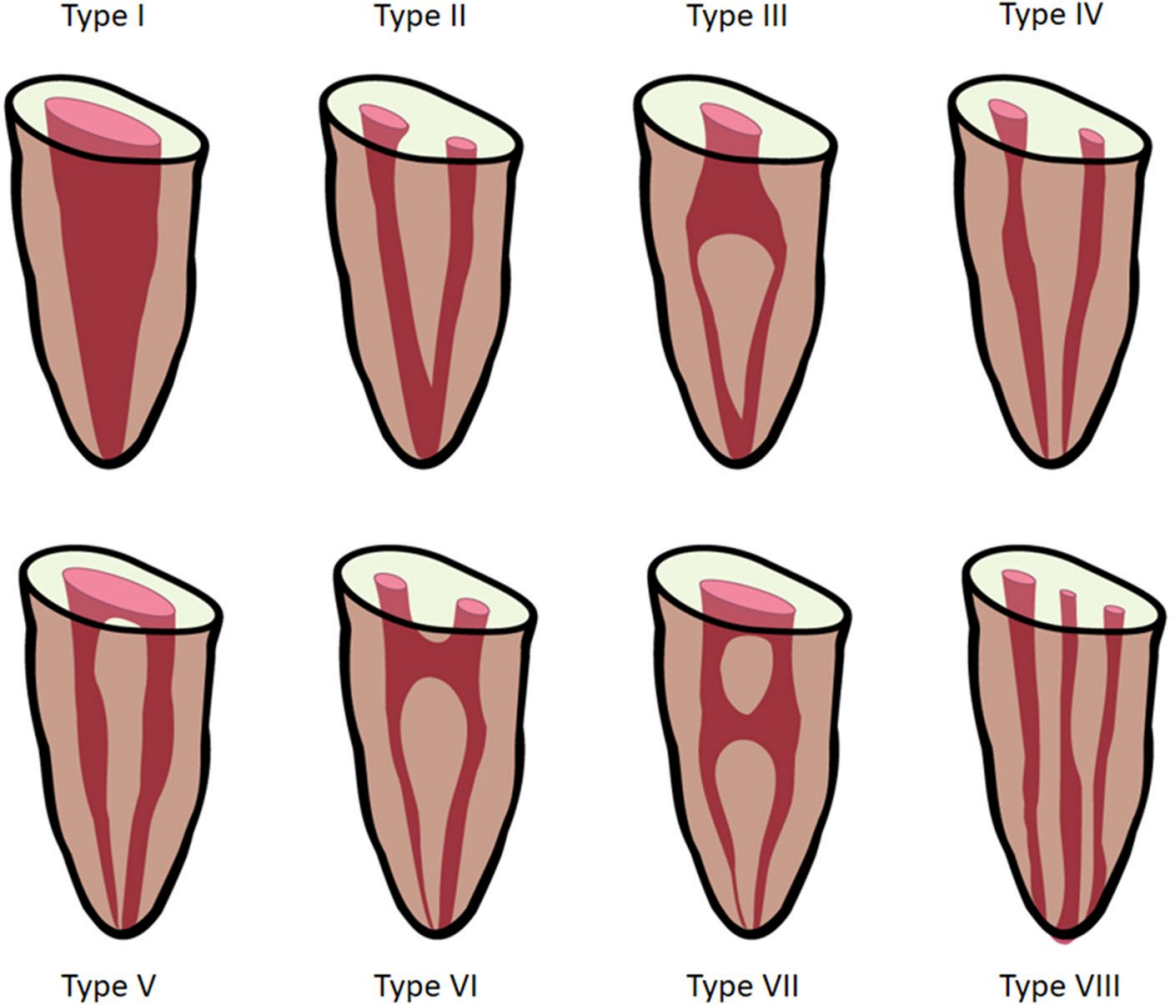
Vertucci’s root canal configuration from pulp chamber to the root apex.

In the literature, there have been reports of variants for each tooth in permanent dentition^15^. Because it is believed that racial and genetic factors influence root canal configuration, different ethnic populations exhibit varied root canal morphologies^16^. The most usual morphology of permanent mandibular incisors (MIs) is a single root with a single root canal^16^ (Fig. 3). However, the root canal morphology of permanent MIs is intricate by the existence of a second canal, lateral canals, and apical deltas, and is not easy to identify^17^. However, studies have revealed considerable variations in the morphology of the mandibular anterior teeth's root canal system^18–21^. The study conducted by Rankine et al. in the Australian population indicated a high prevalence of two canals in the MIs^22^, stimulating further research on the canal configuration of other teeth.

The root canal architecture can be studied using ex vivo methods, performed on extracted teeth, or in vivo methods, performed clinically on patients^23^. The majority of the studies have been conducted in vitro on extracted teeth using invasive techniques such as canal and modified canal staining, tooth-clearing procedures^24,25^, transverse cross-sectioning^26^, scanning electron microscopy^27^, and stereomicroscopy^28^. Radiography is one of the most essential clinical tools for understanding the internal canal anatomy during endodontic therapy. However, radiography uses two-dimensional (2D) projections of three-dimensional (3D) objects; thus, traditional periapical radiography has certain limitations, such as root distortion and superimposition by surrounding structures^29–31^. Cone-beam computed tomography (CBCT) is regarded as more accurate than conventional radiography and as accurate as the modified canal staining and tooth clearing techniques in detecting root canal systems. CBCT provides 3D images of the teeth and surrounding tissues in three orthogonal planes^32^. In addition, compared with traditional computed tomography, CBCT delivers a substantially lower effective radiation dosage and is a non-invasive procedure that produces high-quality images.

Although the structural symmetry of root morphology and the root canal configurations between the teeth on both sides of the jaws are of high clinical significance when treating two opposite teeth in the same patient, a study by Wu et al. found a positive correlation in Taiwanese population, also similar findings reported in Cantonese population, but no extensive studies exist on this topic^33,34^. To the best of our knowledge, information regarding the symmetry and distribution patterns between permanent MFMs with DLRs and permanent MIs with complicated root canal configurations, ipsilaterally and contralaterally among different age groups and sex, using CBCT images has not been reported before in the Indian population.

Therefore, the aim of this study was to observe the prevalence of DLR in the permanent mandibular first molar and complicated canal morphology in permanent MIs in different age groups and sex and to examine whether any association exists between the two in the Indian population using CBCT.

## Materials and methods

The research was carried out in accordance with guidelines and regulations set down in the declaration of Helsinki. The current study was approved by the institutional ethics committee of the Nair Hospital Dental College, Mumbai (EC/PG-10/CONS/2019 dated 22/10/2019). The informed consent was waived by the institutional ethics committee of the Nair Hospital Dental College, Mumbai. The CBCT scans were saved in the Digital Imaging and Communications in Medicine (DICOM) format, and these data were saved in an encrypted file confidentially protected yet retrievable if needed.

### Sample size calculation

The study sample size was determined based on the expected proportion of findings in the control values estimated from the literature^35^. Approximately 400 patients/scans were required in the present study, yielding 800 pairs of observations.

### Sample selection

CBCT images of the mandibular arch collected from patients who required CBCT scans as part of their treatment were evaluated. The indications for CBCT scanning include the assessment of bone volume for dental implant planning, diagnosis of dentoalveolar trauma, management of impacted teeth before orthodontic treatment, and treatment planning before non-surgical and surgical endodontic treatment.

All of the CBCT scans available from the archives were evaluated retrospectively; based on the inclusion criteria, 400 CBCT scans involving the entire mandibular arch, yielding 800 observations, were included in the study.

Of the 1000 patient CBCT images initially scanned, 400 patients (800 MIs and 800 MFMs) were eligible for further analysis. Of these subjects, 126 (38.5%) were females and 274 (68.5%) were males, with an average age of 44.82 ± 14.89 years (203 samples less than 44 years and 197 above 44 years of age).

### Criteria

#### Inclusion criteria

1. Permanent MFMs present bilaterally with complete root formation.
2. Permanent mandibular incisors present bilaterally with complete root formation.
3. Availability of high-quality CBCT images in which the canal orifice and canal configuration can be easily recognized.

#### Exclusion criteria

1. Endodontically treated teeth.
2. Presence of coronal or post-coronal restoration.
3. Presence of root resorption/periapical lesions/calcification/open apices.
4. CBCT scans having artefacts/poor resolution.

#### Image acquisition

The evaluated CBCT images were acquired using a CBCT scanner (PLANMECA PROMAX 3D MACHINE) at 90 kV and 10 mA with an exposure time of 27 s. The voxel size of the images was 0.2 mm, and the slice thickness was 0.1 mm. The acquisition process was performed by a licensed radiologist, an expert in operating and acquiring CBCT images according to the manufacturer’s recommended protocol with the minimum exposure necessary for adequate image quality. The “as low as reasonably achievable” protocol was followed.

#### Image evaluation

The CBCT images were analyzed using Planmeca Romexis imaging software. To ensure optimal visualization, the brightness and contrast of the images were adjusted using the image processing tool embedded in the software.

Two observers, who were endodontists with more than 10 years of experience in the subject, and also experienced in analyzing CBCT images, examined the scans. In order to avoid eye strain, breaks were planned to follow the evaluation of 5 scans, and more than 5 scans were not examined in a row. Inter-examiner bias was resolved by the same observers by analyzing the scans twice within a span of 24 h. The kappa analysis was performed on 10% of total data that is 40 samples before disagreements among examiners were discussed and resolved. There was a near-perfect agreement between Observer 1 and 2 for the presence of Distolingual Root on Right and left side; Vertucci Classification of Central and lateral incisor on the right and left side, (*p* < 0.05) (Table 1).

**Table 1:** Inter-rater reliability.

Inter-rater reliability between observer 1 and 2	Kappa	*p* < 0.01
Presence of distolingual root right	0.848	
Presence of distolingual root left	0.802	
Vertucci classification right central incisor	0.978	
Vertucci classification right lateral incisor	0.968	
Vertucci classification left central incisor	0.969	
Vertucci classification left lateral incisor	0.976	

The following information was recorded and analyzed:

1. The number of roots for permanent mandibular first molar on both sides.
2. The root canal configuration [according to Vertucci’s classification FJ^16^ (Fig. 3) for permanent mandibular central and lateral incisors].

All qualified permanent mandibular incisor and mandibular first molar images were morphologically studied in detail using Planmeca Romexis imaging software.

### Mandibular first molar with DLR (RE)

A series of images were assessed from the crown down to the apex in axial sections to identify the presence of DLRs in permanent MFMs.

The scans were further categorized based on the presence or absence of DLRs as follows:

1. *Non-DLR* No DLR was found in the MFMs on either side.
2. *Unilateral DLR (Uni-DLR)* ADLR was found in one mandibular first molar on either the left or right side, and no DLR was found in the other first molar on the contralateral side.
3. *Bilateral DLR (Bil-DLR)* DLRs were found in both the right and left MFMs.

### Root canal morphology of permanent MIs

A total of 400 scans were assessed to evaluate the root canal morphology of the permanent mandibular central (n = 800) and lateral (n = 800) incisors on the right and left sides. The root canal configuration of the mandibular central and lateral incisors was determined according to Vertucci’s classification. Several cross-sectional images from the cementoenamel junction to the root apex of the mandibular central and lateral incisors were studied to evaluate the anatomy of the root canals, and the canal configuration was categorized as follows:

1. *Simple* The presence of one root and one canal in the MIs was categorized as a “single canal.”
2. *Complicated* The occurrence of more than one root and canal in the MIs was categorized as a “complicated canal.”

In their respective study on mandibular premolars and mandibular incisors, Liu^36^ and Wu ^35^ have categorized Vertucci’s two canal as complicated canal morphology.

The different levels to detect canal morphology was based on the study by Kurumboor K. In this study, the average root length of mandibular central incisor was 12.9 mm and lateral incisor was 12.83 mm. Based on this findings, each section was studied at three levels i.e. 3 mm from apex to study apical third, 6 mm from apex for middle third and 9 mm from apex for coronal third, all three studied in axial section of CBCT scans^37^.

The root canal morphology of the mandibular central and lateral incisors was assessed in sagittal sections at three different levels: 3 mm from the radiographic apex to assess the morphology in the apical third; 6 mm from the apex to assess the morphology of the canals in the middle third of the root; and 9 mm from the apex to assess the morphology of the canals in the coronal third of the root.

The canal configuration and the numbers of the roots and canals of the central and lateral incisors were classified according to Vertucci’s classification^16^.

### Statistical analysis

The data was obtained and entered in Microsoft Excel Version 13. Using IBM SPSS Version 21, statistical analysis of the data was performed. The Data was observed to be categorical for multiple variables hence frequency and proportion was obtained for continuous variable like age; Mean and SD was obtained. Descriptive statistics were expressed as means and standard deviations, frequencies, or percentages, as appropriate, of each measurement calculated at the subject and/or tooth levels. The chi-square test was used for examining differences in categorical variables such as side (left vs right), age (44 ± 52 years) and gender (male vs female) with DLR group (Non-DLR, Uni-DLR, or Bil-DLR). To compare between Gender and Vertucci Classification and Age Categorization and Vertucci Classification, Mann Whitney U test was applied. The association between the prevalence of DLRs and the complex root canal morphology of incisors were analyzed using the chi-square test and SPSS 17.0 software (SPSS Inc, Chicago, IL). All the statistical tests were applied keeping confidence interval at 95% and (*p* < 0.05) was considered to be statistically significant.

## Results

### Prevalence of DLRs

**Figure 4:**
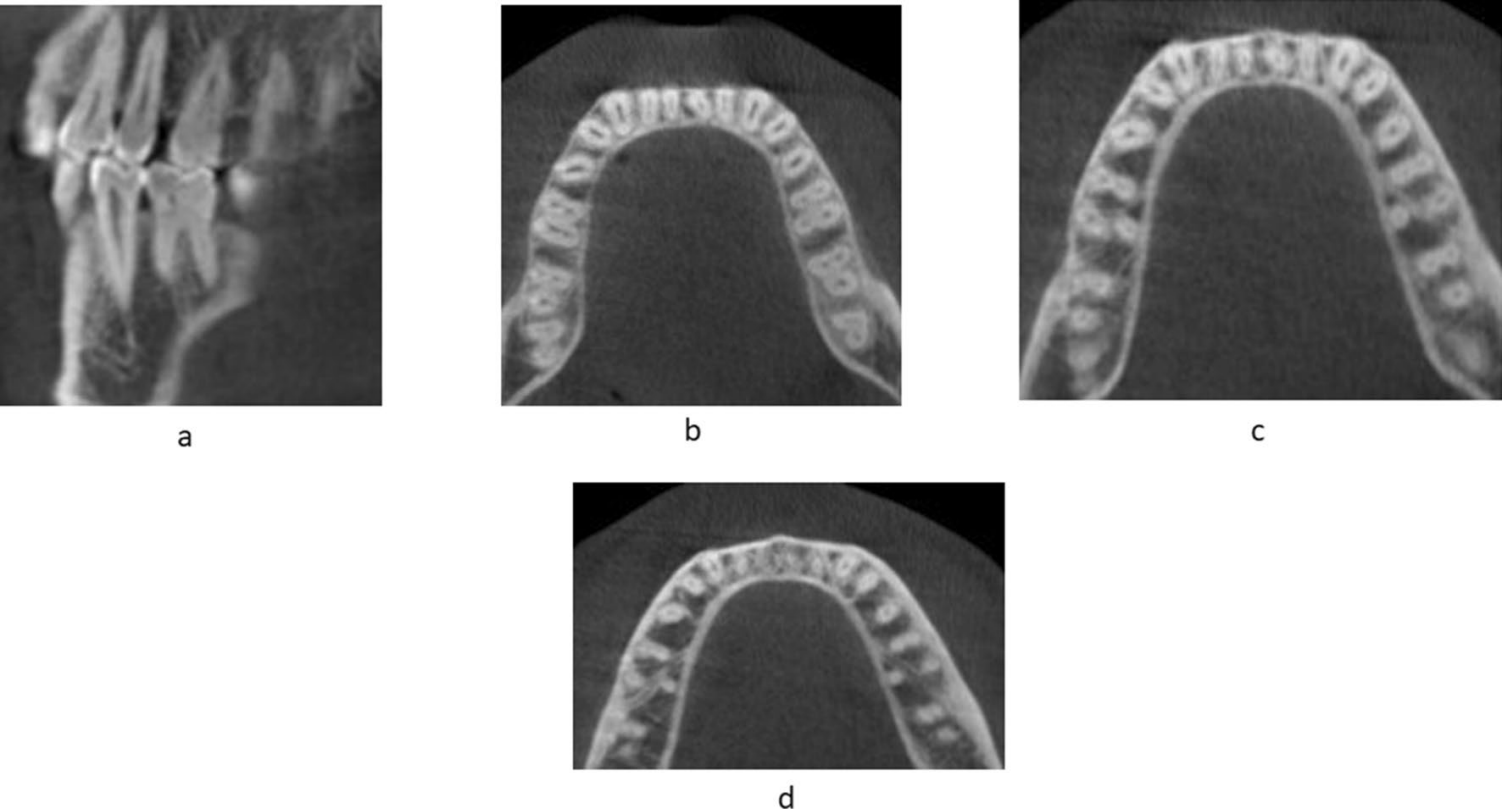
CBCT scans showing DLR in MFMs through various sections from coronal to apical. (**a**) Sagittal section of mandibular first molar. (**b**) Coronal third section of the axial view. (**c**) Middle third section of the axial view. (**d**) Apical third section of the axial view.

**Figure 5:**
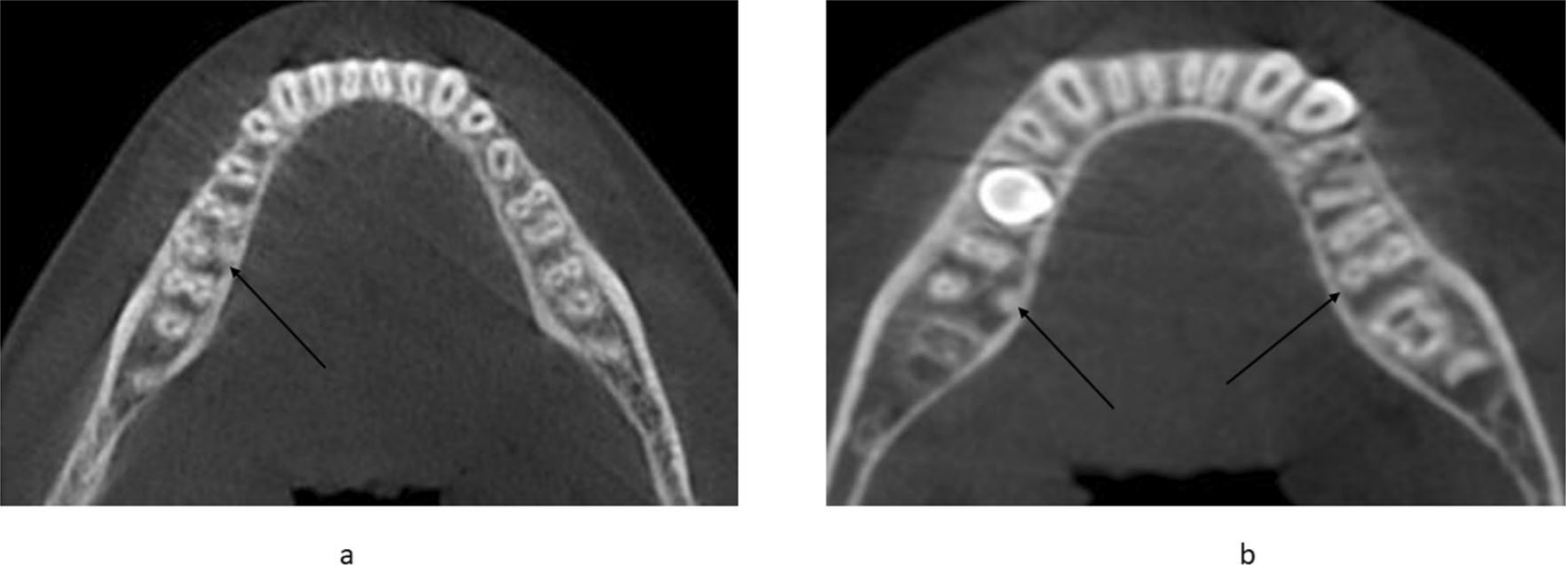
CBCT images showing (**a**) Unilateral and (**b**) Bilateral distribution of DLR in MFMs.

A total of 400 scans exhibiting 800 MFMs were assessed for the presence of DLR. Of the 800 MFMs evaluated, 6.62% (53 of 800) exhibited DLR. Of the total 800 MFMs evaluated, 7.8% (31 out of 400) right and 5.5% (22 out of 400) left MFMs exhibited distolingual roots (*p* > 0.01)(Table 2) (Fig. 4). Of the total 400 scans examined, 3.5% (14 out of 400) showed the unilateral presence of DLRs. However, the prevalence of bilateral DLRs in the MFMs was found to be 5% (20 out of 400) (Fig. 5).According to the gender-wise distribution of DLR, 9.5% (38 out of 400) males and 3.75% (15 out of 400) females showed presence of DLR (*p* > 0.05 on right and left) (Table 3). Age-wise distribution of DLR in MFM showed more prevalence in the age group above 44 years on both the right and left side that is 8.5% (34 out of 400) and 4.75% (19 out of 400) in groups less than 44 years (*p* > 0.05 on right and left) (Table 4). There was no statistically significant difference in the distribution of DLR in MFM among age, sex, and side (*p* > 0.05).

**Table 2:** Distribution of presence of Distolingual root.

	Right	Left
Absent	369 (92.3%)	378 (94.5%)
Present	31 (7.8%)	22 (5.5%)
Total	400 (100%)	400 (100%)
*p* < 0.01

**Table 3:** Comparison of Presence of Distolingual Root and Gender.

	Right		Left	
	Absent	Present	Absent	Present
Male	252 (92.0%)	22 (8.0%)	258 (94.2%)	16 (5.8%)
Female	117 (92.9%)	9 (7.1%)	120 (95.2%)	6 (4.8%)
Total	369 (92.3%)	31 (7.8%)	378 (94.2%)	22 (5.5%)
*P* value	0.758		0.661

**Table 4:** Comparison of Presence of Distolingual Root and Age Categorization.

	Right		Left	
	Absent	Present	Absent	Present
Less than 44 Years	192 (94.6%)	11 (5.4%)	195 (96.1%)	8 (3.9%)
More than 44 Years	177 (89.8%)	20 (10.2%)	183 (92.9%)	14 (7.1%)
Total	369 (92.3%)	31 (7.8%)	378 (94.5%)	22 (5.5%)
*P* value	0.077		0.166	

### Root canal morphology of MIs

#### Mandibular central incisors

**Figure 6:**
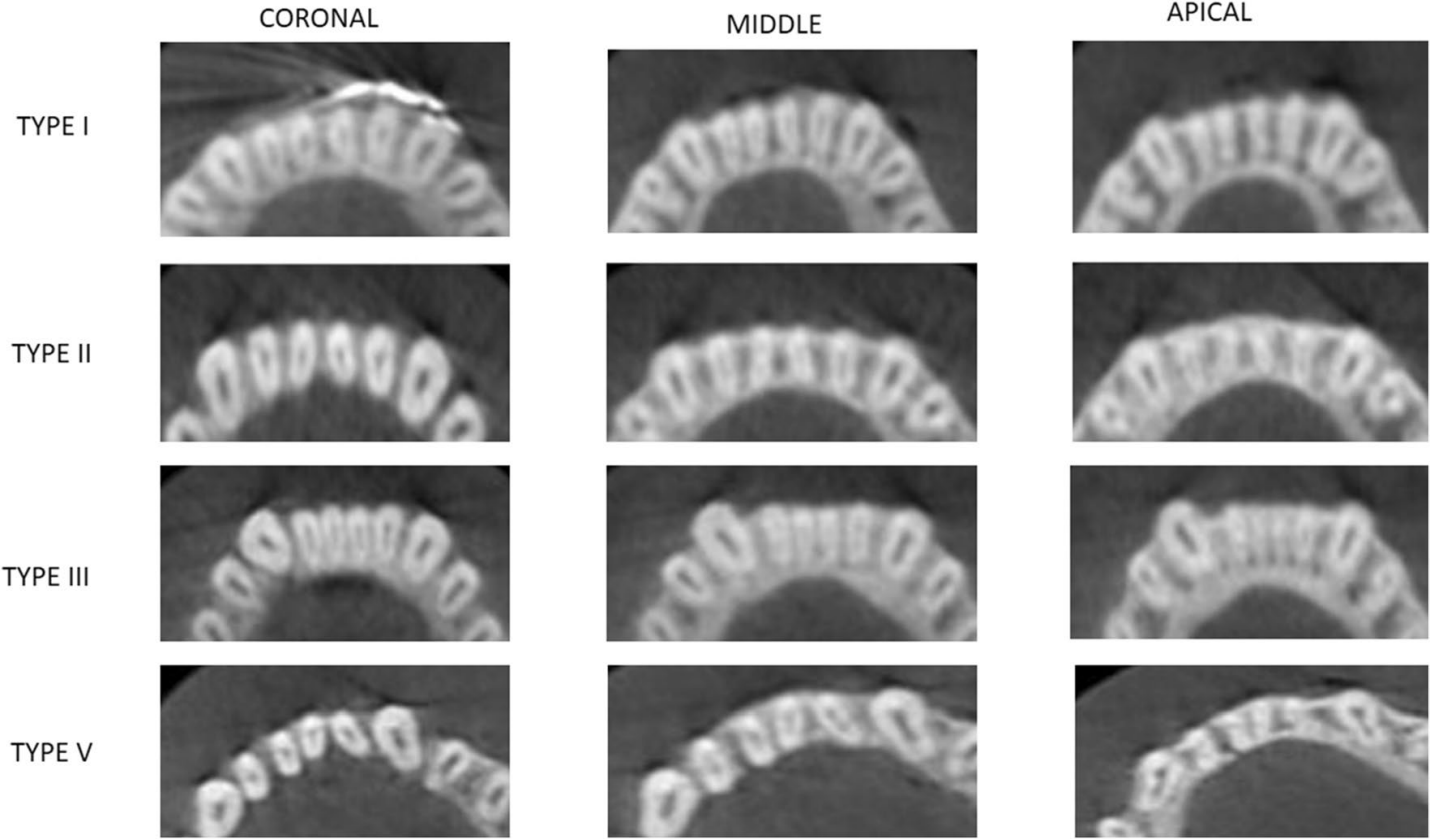
CBCT scans showing root canal morphology according to Vertucci’s classification in Mandibular incisors.

Overall, 66.75% (534 of 800) of the mandibular central incisors showed a simple (Vertucci Type I) root canal morphology. Of the 800 central incisors evaluated, 68% (272 of 400) on the right and 65.5% (262 of 400) on the left side showed a simple canal morphology (Table 5) (*p* > 0.01) (Fig. 6).

**Table 5:** Distribution of Vertucci Classification in Right and Left Central and Lateral Incisors.

Vertucci Classification	Right		Left	
	Central Incisor	Lateral Incisor	Central Incisor	Lateral Incisor
I	272 (68.0%)	233 (58.3%)	262 (65.5%)	236 (59.0%)
II	3 (0.8%)	6(1.5%)	3 (0.8%)	5 (1.3%)
III	113 (28.3%)	139 (34.8%)	124 (31.0%)	143 (35.8%)
IV	0%	1 (0.3%)	0%	0%
V	12(3.0%)	21 (5.3%)	11 (2.8%)	16 (4.0%)
Total	400 (100%)	400 (100%)	400 (100%)	400 (100%)
*p* < 0.01

Approximately 33.25% (266 of 800) of the mandibular central incisors showed a complicated root canal morphology (Type III, V, or II). Complicated root canal morphology including Vertucci’s Type II, III, IV, and V was found in 32% (128 of 400) of MCIs on the right side and 34.5% (138 of 400) of the left side. Vertucci Type III (28.3% on the right side and 31% on the left side) canal configuration was the second most common root canal morphology for the mandibular central incisors, followed by Type V and II (*p* > 0.01) (Table 5).

MCIs, on both the right and left side showed Vertucci’s Type I morphology more commonly in males (66.8–68.6%) than females (62.7–66.8%) (*p* > 0.05 on right and left) (Table 6). Also, the prevalence of complicated root canal morphology of MCIs was more in males than females. Age wise distribution of root canal morphology shows more prevalence of Type I root canal configuration in age groups below 44 years and more prevalence of complicated root canal morphology in age above 44 years on both right and left sides. (*p* > 0.05 on right and left) (Table 7).

**Table 6:** Comparison of Vertucci Classification and Gender.

	I	II	III	IV	V	*P* value
Right Central Incisor						
Male	188 (68.61%)	2 (0.73%)	75 (27.37%)	0%	9 (3.28%)	0.752
Female	84 (66.7%)	1 (0.8%)	38 (30.2%)	0%	3 (2.4%)	
Total	272 (68.0%)	3 (0.8%)	113 (28.3%)	0%	12 (3.0%)	
Right Lateral Incisor						
Male	162 (59.1%)	3 (1.1%)	93 (33.9%)	1 (0.4%)	15 (5.5%)	0.743
Female	71 (56.3%)	3 (2.4%)	46 (36.5%)	0	6(4.8%)	
Total	233 (58.3%)	6 (1.5%)	139 (34.8%)	1 (0.3%)	21 (5.3%)	
Left Central Incisor						
Male	183 (66.8%)	2 (0.7%)	81 (29.6%)	0%	8 (2.9%)	0.461
Female	79 (62.7%)	1 (0.8%)	43 (34.1%)	0%	3 (2.4%)	
Total	262 (65.5%)	3 (0.8%)	124 (31.0%)	0%	11 (2.8%)	
Left Lateral Incisor						
Male	164 (59.9%)	3 (1.1%)	94 (34.3%)	0%	13 (4.7%)	0.778
Female	72 (57.1%)	2 (1.6%)	49 (38.9%)	0%	3 (2.4%)	
Total	236 (59.0%)	5 (1.3%)	143 (35.8%)	0%	16 (4.0%)

**Table 7:** Comparison of Vertucci Classification and Age Categorization.

	I	II	III	IV	V	*P* value
Right Central Incisor						
Less than 44 Years	147 (72.4%)	0	45 (22.2%)	0%	11 (5.4%)	0.150
More than 44 Years	125 (63.5%)	3 (1.5%)	68 (34.5%)	0%	1(0.5%)	
Total	272 (68.0%)	3 (0.8%)	113 (28.3%)	0%	12 (3.0%)	
Right Lateral Incisor						
Less than 44 Years	124 (61.1%)	2 (1.0%)	60 (29.6%)	1 (0.5%)	16 (7.9%)	0.577
More than 44 Years	109 (55.3%)	4 (2.0%)	79 (40.1%)	0	5 (2.5%)	
Total	233 (58.3%)	6 (1.5%)	139 (34.8%)	1 (0.3%)	21 (5.3%)	
Left Central Incisor						
Less than 44 Years	143 (70.4%)	0	50 (24.6%)	0%	10 (4.9%)	0.101
More than 44 Years	119 (60.4%)	3 (1.5%)	74 (37.6%)	0%	1 (0.5%)	
Total	262 (65.5%)	3 (0.8%)	124 (31.0%)	0%	11 (2.8%)	
Left Lateral Incisor						
Less than 44 Years	126 (62.1%)	2 (1.0%)	63 (31.0%)	0%	12 (5.9%)	0.388
More than 44 Years	110 (55.8%)	3 (1.5%)	80 (40.6%)	0%	4 (2.0%)	
Total	236 (59.0%)	5 (1.3%)	143 (35.8%)	0%	16 (4.0%)	

Regarding age, gender, and side, there was no statistically significant variation in the distribution of root canal morphology in MCIs (*p* > 0.05).

#### Mandibular lateral incisors

Overall, 58.62% (469 of 800) of the mandibular lateral incisors showed a simple (Vertucci Type I) root canal morphology. Of the 800lateral incisors evaluated, 58.3% (233 of 400) on the right and 59% (236 of 400) on the left side showed a simple canal morphology (*p* > 0.01) (Table 5) (Fig. 6).

Approximately 41.37% (331of 800) of the mandibular lateral incisors showed a complicated root canal morphology (Type III, V, or II). Complicated root canal morphology including Vertucci’s Type II, III, IV, and V was found in 41.75% (167 of 400) of MCIs on the right side and 41% (164 of 400) of the left side. Vertucci Type III (34.8% on the right side and 35.8% on the left side) canal configuration was the second most common root canal morphology for the mandibular lateral incisors, followed by Type V and II (*p* > 0.01) (Table 5).

MLIs, on both the right and left side showed Vertucci’s Type I morphology more commonly in males (59–59.9%) than females (56.3–57.1%) (Table 6). Also, the prevalence of complicated root canal morphology of MCIs was more in males than females (*p* > 0.05 on right and left). Age wise distribution of root canal morphology shows more prevalence of Type I root canal configuration in age groups below 44 years and more prevalence of complicated root canal morphology in age above 44 years on both right and left sides (*p* > 0.05 on right and left) (Table 7).

Regarding age, gender, and side, there was no statistically significant variation in the distribution of root canal morphology in MCIs (*p* > 0.05).

Association Between DLR and the Root Canal Morphology of Permanent MIs

### In relation to the central incisors

Overall, the prevalence of complicated root canal configuration of the mandibular right and left central incisors in the presence of the first molar with DLR was 32% (128 of 400) and 34.5% (138 of 400) respectively. Approximately 7.8% (31 of 400) and 5.5% (22 of 400) of the permanent MFMs exhibited DLRs on the right and left sides, respectively. In the presence of DLRs, 27.2% (9 of 33) and 30.3% (10 of 33) of the central incisors showed complicated root canal configurations on the right and left sides, respectively. In the absence of DLRs, 32.4% (119 of 367) and 34.8% (128 of 367) of the central incisors showed complicated root canal morphology on the right and left sides, respectively. Therefore, the frequency of complicated root canal morphology in MCIs was more in the absence of DLR in MFMs as compared to the presence of DLR (*p* > 0.05 on right and left) (Table 8).

**Table 8:** Comparison of Vertucci Classification and Presence of Distolingual Root.

	I	II	III	IV	V	*P* Value
Right Central Incisor						
Absent	248 (67.6%)	3 (0.8%)	105 (28.6%)	0%	11 (3.0%)	0.576
Present	24 (72.7%)	0	8 (24.2%)	0%	1(3.0%)	
Total	272 (68.0%)	3 (0.8%)	113 (28.3%)	0%	12 (3.0%)	
Right Lateral Incisor						
Absent	213 (58.0%)	6 (1.6%)	127 (34.6%)	1 (0.3%)	20 (5.4%)	0.748
Present	20 (60.6%)	0	12 (36.4%)	0	1 (3.0%)	
Total	233 (58.3%)	6 (1.5%)	139 (34.8%)	1 (0.3%)	21 (5.3%)	
Left Central Incisor						
Absent	239 (65.1%)	3 (0.8%)	115 (31.3%)	0%	10 (2.7%)	0.637
Present	23 (69.7%)	0	9 (27.3%)	0%	1 (3.0%)	
Total	262 (65.5%)	3 (0.8%)	124 (31.0%)	0%	11 (2.8%)	
Left Lateral Incisor						
Absent	216 (58.9%)	5 (1.4%)	131 (35.7%)	0%	15 (4.1%)	0.859
Present	20 (60.6%)	0	12 (36.4%)	0%	1 (3.0%)	
Total	236 (59.0%)	5 (1.3%)	143 (35.8%)	0%	16 (4.0%)	

**Figure 7:**
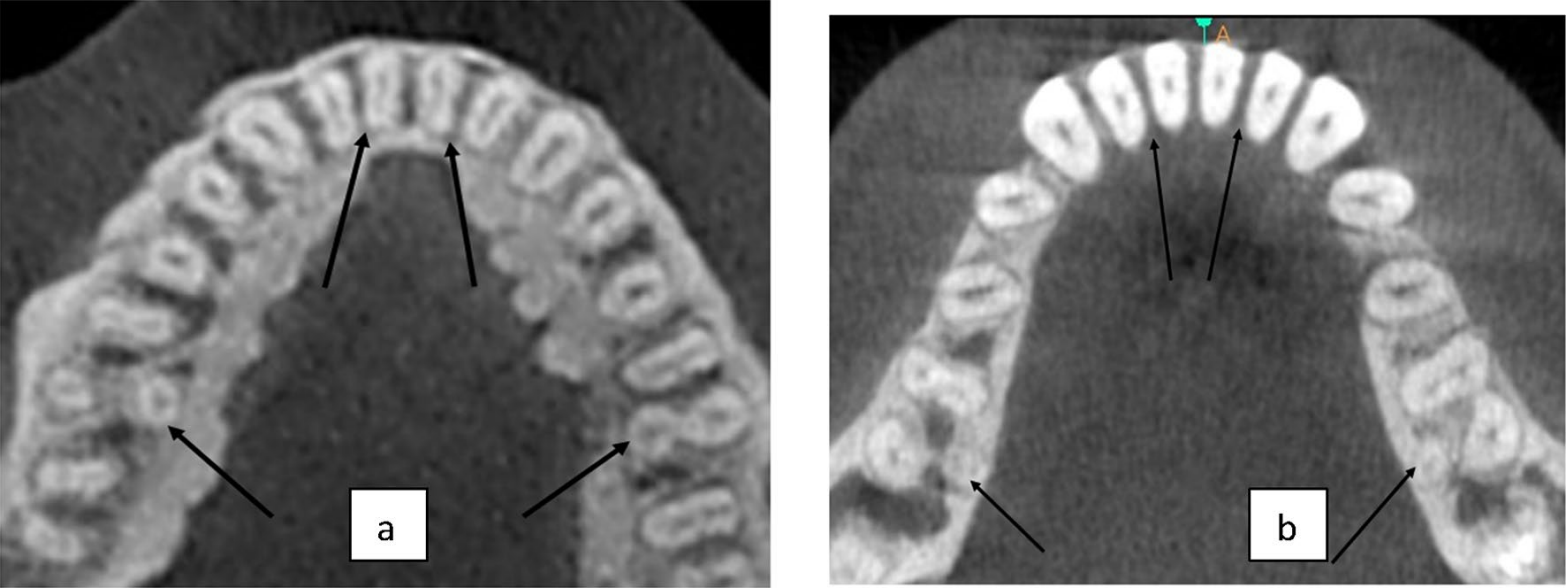
Axial section of CBCT scans showing a) Complicated and b) Simple root canal configuration of Mandibular Incisors in presence of DLR in MFMs.

Since the* p* value is more than 0.05, null hypothesis is accepted that there is no correlation between presence of DLR and complicated root canal anatomy in MCIs (Fig. 7).

### In relation to the lateral incisors

Overall, In the presence of the first molar with DLR, the prevalence of a complicated root canal configuration of the mandibular right and left lateral incisors was 41.75% (167 of 400) and 41% (164 of 400), respectively. Approximately 7.8% (31 of 400) and 5.5% (22 of 400) of the permanent MFMs exhibited DLRs on the right and left sides, respectively. In the presence of DLRs, 39.3% (13 of 33) and 39.4% (13 of 33) of the lateral incisors showed complicated root canal configurations on the right and left sides, respectively. In the absence of DLRs, 41.9% (154 of 367) and 41.1% (151 of 367) of the lateral incisors showed complicated root canal morphology on the right and left sides, respectively. Therefore, the frequency of complicated root canal morphology in MLIs was more in absence of DLR in MFMs as compared to the presence of DLR (*p* > 0.05 on right and left) (Table 8).

Since the* p* value is more than 0.05, the null hypothesis is accepted that there is no correlation between the presence of DLR and complicated root canal anatomy in MLIs.

## Discussion

The comprehension of the macroscopic and microscopic structure of teeth influences the parameters for performing root canal therapy and can have a direct impact on the corresponding likelihood of a positive outcome. Clinicians should be aware of the occurrence and incidence of dental aberrations. MFMs appear to be the teeth that call for root canal therapy the most often since they are the first permanent teeth to erupt in the oral cavity^38^. Nonetheless, many general practitioners experience difficulty in understanding the structural differences in the root canal system in molars, premolars, and incisors.

This study mainly aimed to understand the prevalence of DLR in permanent MFMs and to find out whether any possible association exist of this value with the complicated root canal morphology of permanent MIs in the same patient using CBCT.

In this study, we evaluated the prevalence of DLR in MFMs and the frequency of the Vertucci root canal configuration in MIs and their gender and age influence on its distribution in 400 patients in the Indian population. 800 MFMs and 1600 MIs were evaluated bilaterally. Finally, we evaluated the possible association between the root canal configuration of MIs and the appearance of DLR in MFMs.

In the current study, of the 800 MFMs evaluated, 6.62% (53 of 800) exhibited DLR. More DLRs were present on the right side (7.8%) than on the left side (5.5%) of the first molars; moreover, there was more prevalence of DLRs in males compared to females and in mean age group above 44 years. The distribution of DLRs in MFMs did not differ significantly according to statistical analysis (*p* > 0.05) between males and females or across age groups.

The endodontic treatment of the mandibular anterior teeth and MFMs can be arduous in the presence of additional root canals, which may not be detected by periapical radiographs^33^. Conventional radiography has a significant constraint in that it produces a 2D image of a 3D structure; moreover, superimposing anatomical components may cause the anatomy to be incorrectly interpreted^39^. The presence of DLRs has brought about more challenges for the root canal treatment of MFMs. The DLR canal orifice can easily be overlooked, which may lead to the omission of the DLR canal and result in treatment failure. In addition to the challenges associated with its exploration, the instrumentation and obturation of this additional root have also posed challenges because the root is normally curved^40^. It is generally agreed that when the angle of curvature increases, the chance of instrument fracture rises considerably^41,42^. While instrumenting additional canals, challenges due to anatomy-related issues can arise, including furcal or strip perforation, root weakening, vertical root fracture, root canal flattening and transportation, study length loss, and instrument fractures^43^.

The occurrence of DLR was thought to be a major anatomical variant in the MFMs, and ethnic and geographical factors played an important role in this variation. Chinese^44^ and Taiwanese^7^ populations showed a higher prevalence of DLRs (22% and 33%, respectively), whereas Caucasians^45^, Senegalese^46^, German^47^, and Brazilian^48^ populations showed a lower prevalence (3%–5%).

Various studies conducted using conventional radiographs on Indian populations have shown a prevalence of DLR in MFMs to be 13.3%^1^, 9.1%^49^, 7.67%^50^, 13%^51^, and 13.16%^52^. This finding corroborated our study results, which showed a prevalence rate of 5.5–7.8%, with a higher occurrence on the right side. The variation in the prevalence rate is attributable to the proportion, perhaps because of the different techniques used for analyzing the root canal systems, study design, sample sizes, and geographic and ethnic backgrounds^38^.

Owing to its complicated anatomical variance, the mandibular incisors are one of the most challenging teeth to treat endodontically. In this study, 1600 mandibular anterior teeth from the Indian subpopulation were evaluated to determine the frequency of root canal configurations according to Vertucci FJ. One of the most significant factors in the failure of endodontic therapy for MIs is the presence of an untreated additional canal or missed canal, particularly a lingual canal. If only one of the two existing canals is treated, the pulp tissue of the second canal becomes necrotic and produces toxic agents, which can reach the periodontal ligament through an accessory or lateral canal. The presence of additional canals and bifurcations in MIs requires modifications to the instrumentation technique and obturation. The detection of anatomical complexities on periapical radiographs may be challenging, even for experienced dentists, and may lead to misdiagnosis owing to the 2D nature of periapical radiography.

An ideal technique for the examination of root canal anatomy should be accurate, simple, non-destructive, and usable in vivo. CBCT serves as an excellent imaging method that helps detect the external and internal tooth anatomy. CBCT is currently used widely in implantology, maxillofacial surgery, and endodontics. Moreover, CBCT is useful for the diagnosis of root canal anatomy^53,54^ and for identifying additional canals and roots in teeth^55,56^. Compared with conventional CT, CBCT provides better-quality images for the assessment of dental hard tissues with considerably reduced radiation exposure. Compared with conventional intraoral radiographs alone, CBCT is an excellent tool that helps provide additional information for diagnosis and enables more predictable management of complicated endodontic problems. Furthermore, CBCT images can be analyzed using simple, manufacturer-designed software that does not interfere with the original format of the images, thereby permitting the analysis^32^.

In the current study, the mandibular central and lateral incisors on both the right and left sides showed predominance for Vertucci Type I configuration. Vertucci Type I canal configuration showed the highest occurrence (58–70%), followed by Type III (26–35%). These findings corroborate those of the studies conducted by Kaffe et al.^18^, Verma et al.^19^, Rahimi et al.^20^, Saati et al.^21^, Han et al.^55^, and Liu et al.^36^. In an Indian subpopulation, Verma et al. found that the most common canal configuration was Vertucci’s Type I, which was then followed by Type III (15.25%), II (12.12%), V (3.12%), and Type IV (2.37%). Compared with mandibular central incisor, mandibular lateral incisors showed a higher prevalence of having more than one root canal. This finding corroborates those of previous studies that have shown that when compared to other mandibular anterior teeth, the lateral incisors have a greater incidence of multiple root canals. In our study, complicated root canal morphology was found more commonly in the age group more than 44 years which was contrary to that found in the Cantonese population. There was no statistically significant difference between root canal anatomy and side, age or gender (*p* > 0.05).

Although the anatomic symmetry of the root morphology and the root canal configurations between the teeth on the right and left sides in the same patient are of great clinical significance when treating two opposite teeth in the same patient^33,34,57^. Wu et al., in his extensive studies addressed the possible correlation between the prevalence of DLR in PMFMs with the morphologic characteristics of neighboring teeth that is mandibular premolars and mandibular incisors. In these studies, the prevalence of a DLR in MMs was correlated to a higher incidence of complex canal morphology in permanent mandibular central incisors^35^, lateral incisors^58^, and premolars^59^. Similar study has been conducted in Cantonese population which showed positive correlation between anatomies^33^. No such comparative studies have been conducted in Indian population. In our study, we attempted to find out the prevalence of MFMs with DLRs and then correlate it with the root canal morphology of mandibular incisors. In our study, the presence of MFMs with DLRs was not significantly associated with the complicated root canal configuration in MIs. In other words, a significant association did not exist between the MFMs with DLRs and MIs with complicated root canal configurations on both sides (*p* > 0.05).

Clinicians can benefit from our study’s findings on the morphology of MIs and the prevalence of DLRs in MFMs according to the age group, gender and side wise distribution. Association between the complicated root canal configurations of various teeth, particularly the bilateral symmetry rate of DLRs in MFMs and the high levels of complicated canal configurations in MIs, and a possible association between the two can be justified through this study. CBCT has many advantages over other experimental methods to evaluate roots and root canal morphology, such as clearing and staining, sectioning, conventional radiographs, etc.

## Limitations

Other teeth should have been included to find potential association. Larger sample size would have yield better results.

## Conclusion

Our study showed that when DLRs were present in the MFMs in the Indian population, the possibility of complicated root canal configuration in the MIs was lower. Moreover, besides being aware of the changes in the number of roots and root canal configurations, clinicians should be aware that the anatomical differences between the teeth may vary and potential correlation can always be found.
